# Autonomy-connectedness mediates sex differences in symptoms of psychopathology

**DOI:** 10.1371/journal.pone.0181626

**Published:** 2017-08-03

**Authors:** Marrie H. J. Bekker, Marcel A. L. M. van Assen

**Affiliations:** 1 Department of Clinical and Medical Psychology, Tilburg University, Tilburg, the Netherlands; 2 Department of Methodology and Statistics, Tilburg University, Tilburg, the Netherlands; Technion Israel Institute of Technology, ISRAEL

## Abstract

**Objectives:**

This study aimed to examine if autonomy-connectedness, capacity for self-governance under the condition of connectedness, would mediate sex differences in symptoms of various mental disorders (depression, anxiety, eating disorders, antisocial personality disorder).

**Method:**

Participants (*N* = 5,525) from a representative community sample in the Netherlands filled out questionnaires regarding the variables under study.

**Results:**

Autonomy-connectedness (self-awareness, SA; sensitivity to others, SO; capacity for managing new situations, CMNS) fully mediated the sex differences in depression and anxiety, and partly in eating disorder -(drive for thinness, bulimia, and body dissatisfaction) and anti-social personality disorder characteristics. The mediations followed the expected sex-specific patterns. SO related positively to the internalizing disorder indices, and negatively to the anti-social personality disorder. SA related negatively to all disorder indices; and CMNS to all internalizing disorder indices, but positively to the anti-social personality disorder.

**Conclusion:**

Treatment of depression, anxiety, but also eating disorders and the antisocial personality disorder may benefit from a stronger focus on autonomy strengthening.

## Introduction

The existence of sex differences in the prevalence of mental disorders is well-known (e.g., [1]). Anxiety- and mood disorders, the most common mental disorders, are generally more prevalent in women than in men; the estimated proportion of prevalence in women versus men is 2:1 [2]. Other mental disorders have an even more unequal prevalence among the sexes. Eating disorders are recently reported to be between 3–7 times more prevalent in women than in men [3, 4]. Antisocial behavior, conduct disorder, and antisocial personality disorder occur more often in men than in women [5]; men are also 10 to 14 times more likely than women to develop life-course-persistent antisocial behavior [6, 7].

Sex differences in mental disorders are consistent throughout the life span, and independent of health care settings (e.g., [2, 8]). The determinants of the unequal prevalence of mental disorders in both sexes are far from established [9, 10]. Like other health-related sex differences, those in prevalence of mental disorders are plausibly due to a complex interplay between sex, gender, and various sets of factors influenced by both. “Sex” is here meant as biologically based being male or female thus due to genetics, hormones etc.; whereas “gender”, also called “femininity” and “masculinity”, encompasses the biopsychosocial meanings that are within a certain culture attributed to biological sex Oakley [11]. These complex relationships have been summarized in a “Multi-Facet Gender and Health Model” [12]. The model describes how (biological) sex and (sociocultural) gender contribute to sex differences in health including in mental disorders’ prevalence while these relations are moderated and mediated by sex- and gender-affected, adverse, social or interpersonal factors (i) (or: stressors, e.g., sexual violence), (ii) person-related (or: vulnerability) factors (e.g., rumination tendency), and (iii) types of gender bias in (mental) health care (e.g., crying is an indicator for depression but occurs, in general, more in women than in men).

Part of the sex differences in mental disorders’ prevalence may thus be due to sex bias in diagnostics (e.g., [13–15]), such as the tendency to focus on (sex) differences rather than on similarities [16], or to use sex-stereotyped definitions of mental disorders (e.g., [17]). Despite of these types of biases influencing reported figures and statistics, the literature is consistent in accepting the existence of sex differences in mental disorders’ prevalence as facts (e.g., [8, 9, 18–20]).

In the present study we focused on explanations of sex differences in occurrence of mental disorders other than possible sex bias in statistics and diagnostics, and sex differences in stressors. Our study concerned a specific person-related factor that most probably originates from, for both sexes different, attachment experiences and that is closely connected to gender identity. We examined the prevalence of symptoms of depression, anxiety, eating disorders (bulimia, drive for thinness and body dissatisfaction) and the anti-social personality disorder, as these mental disorders are known to have unequal prevalence among men and women. Building on previous research and theory we focused on the role of autonomy in explaining these sex differences. That is, our main goal was investigating whether individual differences in autonomy could at least partly explain the sex differences in these disorder indices. We will, below, first introduce our concept of autonomy-connectedness, followed by a discussion of common explanations of sex differences in mental disorders’ prevalence and to what extent the autonomy-connectedness paradigm might agree with these more well-known explanations. Thereafter, we will present our study aims and design in more detail.

### Autonomy and autonomy-connectedness

Autonomy-Connectedness is an innovative, gender-sensitive autonomy concept, based on attachment theory [21, 22] and neo-analytical object-relation theory with respect to gender-identity [23], It reflects the capacity for self-governance under the condition of connectedness, a psychological capacity to be reached at the beginning of adulthood. The classical concept of autonomy one-sidedly emphasized independence and separation [24–26], not necessarily reflective of healthy psychological functioning. That is, healthy autonomous functioning also presumes the ability to initiate and maintain meaningful relationships, agreeing with and recognizing an aspect of adult psychological functioning, connectedness, more frequently expressed by women [27–29]. This perspective on autonomy was also supported by Hmel and Pincus [30] who, based on a critical review on various autonomy concepts, concluded that autonomy is best conceptualized as “self-governance” characterized by interpersonal connectedness, interdependency, self-awareness and self-insight. Therefore, Bekker and van Assen [28] added the term connectedness to, and integrated it into the autonomy concept. Particularly reflective of this connectedness is one of the three AC components Sensitivity to (the wishes, opinions, and needs of) others; SO. Including this SO makes the concept applicable to both men and women. Notably, women compared with men consistently show much higher SO [27, 28, 31, 32]. The other two components of AC are Self-awareness (SA; the capacity to be aware of one's own opinions, wishes, and needs, and the capacity to express these in social interactions), and Capacity for Managing New Situations (CMNS; (un-)easy feelings in new situations, flexibility, an inclination to exploration, and dependence on familiar structures). CMNS was derived from the capacity for exploring the environment, e.g., as expressed by securely attached children during the Strange Situation Test [33].

The Autonomy–Connectedness Scale (ACS-30; [27, 28]) is a 3-factor scale based on this more modern concept of autonomy and represents, with its three subscales, SA, SO, and CMNS, the three theoretically derived components of autonomy-connectedness [27]. Autonomy and autonomy-connectedness have been shown to be associated with (indices of) various types of psychopathology in several studies, most of them concerning clinical patients. Low autonomy was found related to depression (e.g., [34–38]) and anxiety disorders (e.g., [39]). Studies conducted with the ACS-30 revealed the same pattern. In these studies, depression, anxiety and eating disorders were associated with high sensitivity to others together with low self-awareness (e.g., [40–42]). Regarding personality disorders, being overly sensitive to others contributed to internalizing personality disorders, among others characterized by inhibition of other-directed anger [43]. However, low sensitivity to others as well as high capacity for managing new situations were associated to antisocial personality disorder characteristics [43].

Autonomy-connectedness components appeared to be relatively independent from well-known global personality factors such as the Big Five [32]. Moreover, autonomy-connectedness components were only moderately associated with insecure attachment styles (e.g., [41, 42]), for which, also, associations have been established with many mental disorders including those under study (anxiety and mood disorders, see, e.g., [44]; eating disorders, see, e.g., [45, 46]; and personality disorders, see e.g., [47]). These findings seem to support the idea that, agreeing with attachment theory, insecure attachment experiences imply poor autonomy development and poor autonomy strongly increases the risk for psychopathology. To summarize, these findings suggest that autonomy-connectedness uniquely contributes to various types of psychopathology and milder types of unwell-being and should be considered a separate attachment-related multidimensional construct (e.g., [41]).

One of the most noteworthy findings with the ACS-30 is that men and women consistently differed in levels of sensitivity to others, with women reporting much higher sensitivity to others than men (Cohen’s *d* = .90, strong effect). Concerning Self-awareness (Cohen’s *d* = .23, small effect) and Capacity for Managing with New Situations (Cohen’s *d* = .18, small effect) men scored, in a representative large sample, higher than women (e.g., [31]). The findings that men and women differed both in autonomy and in prevalence rates of mental disorders, suggest that sex differences in autonomy may, to a substantial degree, explain the unequal prevalence rates of these disorders among men and women. This assumption is further supported by findings from clinical studies indicating sex-specific patterns in the associations between autonomy-connectedness and types of psychopathology. In disorders such as anxiety disorders and depression poor autonomy-connectedness appeared to consist of low self-awareness as well as low capacity for managing new situations, together with high sensitivity to others [40, 41]. In the antisocial personality disorder, in contrast, extremely low sensitivity to others was found [48]. Another example is, that high sensitivity to others related negatively to passive-aggressive behavior in women, and prevented them from displaying anger evoked by being treated unfairly [49]; in men, however, sensitivity to others was positively associated with passive-aggressive behavior, and seemed to prevent them from antisocial behavior [48]. Before outlining our specific research hypotheses we will summarize existing explanations for sex difference in prevalence rates in disorders. At the same time, we will argue that these explanations are compatible with an explanatory model in which poor autonomy with its sex-specific manifestations serves as a general risk factor for the mental disorders with unequal prevalence among the sexes.

### Common explanations on sex differences in prevalence of mental disorders and their fit with the autonomy-connectedness perspective

In identifying determinants of sex differences in the prevalence of mental disorders the literature often used the broader categories of internalizing and externalizing mental disorders (e.g., see [10, 43, 50, 51]. Anxiety-, mood-, and eating disorders are typically internalizing disorders, characterized by self-blame, insecurity, self-silencing and inhibition of other-directed anger. Anti-social personality disorder and conduct disorder belong to the group of externalizing disorders. These disorders are characterized by impulsive and often overt dis-inhibited, aggressive behavior (e.g., [18, 19]). The internalizing disorders occur more often in women, whereas the externalizing ones have higher prevalence in men [18, 19].

A prominent explanation of the sex difference using the internalizing and externalizing distinction is the sex role perspective, or, a more specific variant, the gender role stress theory (see [52, 53]). According to this perspective, sex roles in society contribute to internalizing disorders in women and to externalizing disorders in men. Society would prescribe women to “silence their selves”, and discourages assertive behavior as being non-feminine; for men, the sex roles prescribe dominance and discourage weak or vulnerable behavior, such as crying and showing fear or uncertainty. From the gender role stress perspective, the stress resulting from failing to meet masculine or feminine gender role standards, respectively, results in psychopathology. Why such psychopathology manifests in internalizing versus externalizing pathological behavior, such as women’s “silencing” themselves by self-blame and guilt feelings resulting in depression (e.g., [54]) and men’s “controlling” behavior to women by physical aggression [55] remains rather unclear from the gender role stress perspective; the focus is here on the risk factor side: primary being the failure of meeting the respective gender roles. Sex role -, including gender role perspectives are in line with the different sensitivity to others profiles within female and male manifestations of autonomy-connectedness. Women would be (demanded to be) more nurturing, inclined to please others and to inhibit aggression, whereas men would be more inclined and demanded to assert themselves, even against other persons’ interests.

Other more disorder-specific explanations focus on risk factors for specific mental disorders. Regarding depression, one of the most prevalent mental disorder, rumination is a well-established one. Rumination occurred more in women than in men, and may completely mediate the sex differences in depression prevalence (e.g., [56]). Three specific beliefs accounted for the sex difference in rumination, namely beliefs in terms of responsibility for the emotional tone in relationships, loss of mastery over negative events, and loss of control over one’s own emotions (e.g., [56]). The relationships between rumination and autonomy-connectedness have not yet been examined, at least to our awareness. However, one may expect that feeling relational responsibility is associated with sensitivity to others, and loss of mastery over negative events and one’s own emotions with low self-awareness and capacity for managing new situations. Thus, the explanation of the sex difference in depression by rumination may also be in line with an explanation based on autonomy-connectedness.

In explanatory models for the unequal prevalence of anxiety disorders among men and women, the focus primarily laid on the aforementioned sex role socialization processes. Chambless and Mason [57] and McLean and Anderson [58] made plausible that gender socialization processes promote worry, sensitivity to socially transmitted information, and avoidant coping in women, and discourage women to show masculine assertiveness. In this manner, society would breed anxiety disorders in particularly women. The fact that anxiety disorders are generally associated with a general lack of experienced self-efficacy or competence to satisfactorily manage one’s own life, including one’s relationships with others, fits well with the explanation that anxiety may originate from poor autonomy-connectedness [20, 59].

Regarding eating disorders, disorder-specific, sex-related risk factors have been well-established and integrated into Stice’s Dual Pathway Model [60, 61]. Two main factors in the model are thin-ideal internalization and negative affect (e.g., [60]). Thin-ideal internalization, the tendency to internalize societal norms regarding one’s outward appearance, was related to sensitivity to others, namely specifically to the opinions of others, as well as to low self-esteem (e.g., [62, 63]), which is conceptually related to low self-awareness. The same is true for negative affect, which is, more generally, associated to depression with its relation to autonomy-connectedness already described above. The well-known affect regulation model [64], explaining how negative affect can be released by binge eating, core symptom of many eating disorders, further underlined the plausibility of autonomy-connectedness as an explanatory factor. (Negative) Affect regulation by binging was found associated with low interoceptive awareness and alexithymia [61], the latter having been found closely related to poor autonomy [42, 48, 65]. It was well conceivable that emotional eating and binge eating result from the confusion of needs related to negative affect, e.g., the need for being comforted or to express one’s anger, with needs related to food, thus appetite or hunger [61, 66], and that a low level of self-awareness of one’s own needs and emotions is at the core of such confusion.

Regarding the antisocial personality disorder, Cale and Lilienfeld [67] concluded from their review that researchers had only just started research beyond sex differences in the base rates and general symptoms of this behavior. Eme [68], from his review, concluded that the most consistently identified risk factor for (antisocial behavior related) violence was being male. In addition, Lahey et al. [69] emphasized that “the magnitude of differences in levels of antisocial behavior… is so large that a theory of the origins of antisocial behavior that does not explain sex differences would be incomplete”. Eme [68] reviewed the explaining power of some major, sex-specific neuro-developmental variables for the strong association between being male and life-course-persistent (LCP) antisocial behavior, and argued that LCP is best viewed a neuro-developmental disorder. Interestingly, the review also highlighted the huge sex differences in empathy that seem particularly relevant for the LCP subgroup with callous (e.g., lacking empathy, guilt) and unemotional (e.g., shallow emotions) traits (CU; see also [70, 71]). This is in line with the findings of Baron-Cohen [72, 73] and Zahn-Waxler et al. [17] who showed that early preschool girls compared with boys exhibited more empathy and understanding of others' emotions, problems and intentions. The relative absence of fear and empathy in antisocial behavior together with sex differences in this absence is compatible with an autonomy-connectedness perspective relating antisocial behavior to lower self-awareness and particularly lower sensitivity to others. Low sensitivity to others, lack of fear (for others), and low empathy may result from early sex-specific attachment experiences and responses. Boys might be more at risk to develop these characteristics due to more masculine, avoidantly attached coping.

**Fig 1:**
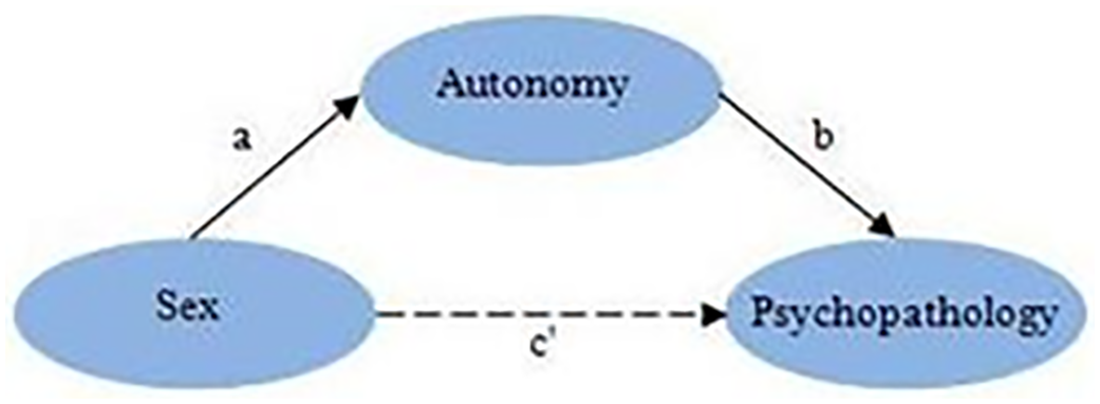
Mediation model. The dashed line indicates that the effect of sex on psychopathology (c) is at least partially explained by the indirect effect via autonomy (a×b). The effect of sex on psychopathology that is not mediated by autonomy is signified by c’.

Taken together, most explanations of the sex differences in prevalence rates of mental disorders agreed with an autonomy approach as explanatory model. We should notice here again that there are also biologically oriented theories regarding these sex differences (see aforementioned Multi-Facet Gender and Health Model). However, due to the general difficulty to translate biological theories into psychological phenomena we choose not to focus on these theories. The compatibility between common explanations of sex differences in mental disorders and the autonomy-connectedness perspective on these sex differences encouraged us to examine to what degree autonomy-connectedness would function as a mediator between being male or female and the unequal sex ratios of various types of psychopathology (Fig 1). We also examined two types of moderation: First, if the effects of autonomy components on mental health indices vary between men and women (which would correspond to an arrow of sex to arrow (b) in Fig 1), and second if the effects of an autonomy component on mental health indices depends on scores on the other two autonomy components (which would be represented by arrows of autonomy component to (b) in Fig 1, had all three components be depicted separately).

### The current study

The main aim of the present study was examining if individual differences in autonomy would at least partially explain sex differences in mental health indices that are known to have unequal prevalence rates among men and women. To this end, we selected mental health indices of those mental disorders that (see above) already proved related to autonomy-connectedness, i.e., anxiety- mood-, eating- and (antisocial) personality disorders. We expected that, after controlling for autonomy, sex differences in (core, DSM IV and 5) symptoms of depression, anxiety, eating disorders (bulimia, drive for thinness and body dissatisfaction) and anti-social behavior would become substantially smaller. That is, we expected that the effect of sex on psychopathology would at least partially be mediated by autonomy. We examined the relationships between autonomy, sex, and mental disorders indices in a large community sample, rather than in relatively small and non-random clinical samples on which much of the now available evidence was based. Notice that using non-random clinical samples can easily lead to selection bias, whereas large representative community samples contain the complete range of mental health indices and autonomy across both sexes.

A second goal was to explore whether the effect of each autonomy component on psychopathology would be moderated by sex, and by each of both other autonomy components. That is, we examined if the effects of autonomy-components on mental health indices were stronger for women than for men, or weaker. And we examined if the effects of an autonomy component (say sensitivity to others) on mental health indices were stronger for lower scores on other autonomy components (e.g. self-awareness and capacity for managing new situations), or weaker. Effects strengthening each other would correspond to synergetic effects or autonomy patterns, i.e. particularly low scores on mental health indices for combinations of scores on autonomy components. More particularly, according to the literature we may expect an interaction of sensitivity to others and self-awareness when predicting internalizing disorders (depression, anxiety, eating disorders), (e.g., [74–77]), i.e. stronger positive effects of sensitivity to others on these disorders for lower self-awareness, as well as when predicting externalizing disorders (antisocial personality disorder) (e.g., [17, 18, 48]), i.e. stronger negative effects of SO on these disorders for higher self-awareness.

## Method

### Participants and procedure

Participants were 5,525 (2,586 male) individuals from a community sample ranging in age from 16 to 93 years (*M* = 48.69, *SD* = 17.12). Of all participants, approximately 24% was single. Half of the participants in a relationship (married or living together) reported to have children. Approximately 33% had a higher educational level, another 33% reported to have a middle educational level, and 29% a lower educational level.

All participants were members of the Liss panel (see http://www.lisspanel.nl). The Liss panel consists of a representative sample of Dutch households spread throughout the Netherlands. Most members reported having Dutch nationality (96.7%; 93.6% were actually born in the Netherlands). Members of this panel are carefully selected by Centerdata, a research institute located at the campus of Tilburg University, in collaboration with Statistics Netherlands (Statistics Netherlands is responsible for collecting and processing data in order to publish statistics to be used in practice, by policymakers and for scientific research. In addition to its responsibility for (official) national statistics, Statistics Netherlands also has the task of producing European (community) statistics). Panel members are paid €15 an hour in exchange for filling out questionnaires on the internet on a variety of subjects once a month. When needed members were provided with a computer and/or internet. On average participants spend 0.5 hours every month filling out questionnaires. Data are collected for scientific purposes only and stored separately from personal name and address information. Participants receive individual passwords (also when living in the same household) to guarantee their privacy. The procedure agrees with the Declaration of Helsinki. During the recruitment of the panel, respondents who agreed to participate in the panel received a confirmation email, and a letter with login code. With the login code provided they could confirm their willingness to participate and immediately start the first questionnaire. This confirmation procedure, following the consent to participate given to the interviewer, ensured the double consent of each respondent to become a panel member and participate in the monthly panel questionnaires. The present study was part of the normal monthly panel questionnaires, for which no specific consents were asked after the general consent for panel participation was given. It has to be noted that ethics approvals for questionnaire research among adults are not required in the Netherlands. In general, CentERdata abides by the Dutch "protection of personal data act" ("Wet Bescherming Persoonsgegevens", WBP), which is consistent with and derived from European law (Directive 95/46/EC). The questionnaire instruction including all items (see below) can be found in S1 Appendix.

### Measures

#### Background measures

Many background measures of the participants are assessed in the Liss panel. Those deemed relevant for our study, which were included in our analyses, were age, relationship (status), and education. Age was calculated using the participants’ ‘year of birth’ at the time of the study. Relationship was assessed with question “Head of the household lives together with a partner (married or not)” (0 = ‘no’, 1 = ‘yes’). Education, originally consisting of nine categories, was coded into 0 = other or unfinished or none, 1 = low (primary school or vmbo), 2 = medium (havo/vwo or mbo), 3 = high (hbo or university).

#### Autonomy-connectedness scale

The Autonomy-Connectedness Scale (ACS-30; [28]) consists of 30 items, divided into three subscales: 7 items for Self-awareness, 17 items for Sensitivity to Others, and 6 items for Capacity for Managing New Situations. Each item can be answered on a 5-point Likert scale ranging from 1 (‘disagree’) to 5 (‘agree’). Examples of subscale items are respectively “Usually it is very clear to me what I like most”, “I often go very deeply in other peoples’ thoughts”, and “I am a very adventurous person”. The scale has a robust three-factor structure, and is known to have good reliability with Cronbach’s α around .80 in previous studies (e.g., [28, 31]). The reliabilities were .75, .78, .74 for Self-awareness, Sensitivity to Others, Capacity for Managing New Situations, respectively, in the present study. The construct validity of the ACS-30 has been confirmed by correlational studies showing associations in the expected directions with relevant scales including the Autonomy subscale of the Personality Research Form ([78]; Dutch adaptation by [79]), the subscales Autonomy, Change, and Succorance of the Adjective Checklist ([80]; Dutch adaptation by [81]); and with self-efficacy at work [28].

#### Beck depression inventory

The Beck Depression Inventory (BDI-II; [82]) measures the frequency of various aspects of depression. The BDI consists of 21 items; each item consists of four different statements receiving a 1–4 score regarding severity of depression. Statements are about several topics, for example sadness, crying, loss of pleasure, self-criticism, self-dislike, and loss of energy. Participants choose between one of the four statements. The scale is widely used and is known to have a good reliability, with Cronbach’s α around .90, and also high concurrent validity (see, for example, [83]). Cronbach’s α was .85 in the present study.

#### The symptom checklist-90

The Hopkins Symptom Checklist-90 (SCL-90; [84]; Dutch version by [85]) contains 8 subscales, of which we used only the Anxiety subscale (10 items). Each of the items refers to a specific complaint, and its seriousness has to be rated on a 5-point scale varying from 1 (‘not at all’) to 5 (‘very much’). Examples of items are ‘Nervousness or shakiness inside’, ‘Suddenly scared for no reason’, and ‘Feeling fearful’. The anxiety subscale generally has a good reliability, with Cronbach’s α = .91 in the present study, and the whole scale appeared to have good structural validity [86].

#### Eating disorder inventory-2

To measure eating disorder symptoms, we used several relevant subscales of the Eating Disorder Inventory (EDI-2: [77]; Dutch translation: [87]). The EDI-2 is a self-rating inventory with 91 items and 11 subscales designed for the assessment of attitudinal and behavioral dimensions relevant to anorexia and bulimia nervosa. In the current research we only used the subscales Drive for Thinness, Bulimia, and Body Dissatisfaction. Examples of the subscales are respectively “I am preoccupied with the desire to be thinner”, “I have gone on eating binges where I felt that I could not stop” and “My thighs are too large”. The Cronbach’s reliabilities of the Drive for Thinness, Bulimia, Body Dissatisfaction scales were .84, .86, and .9, respectively, in the present study. Factor analytical studies supported the construct validity of the scale [88].

#### Anti-social personality disorder

The Questionnaire for Personality Characteristics (Vragenlijst voor Kenmerken van de Persoonlijkheid, or VKP; [89]) consists of 22 subscales and 197 statements, which are based on 9 criteria for personality disorders from the ICD-10 and 13 criteria for all types of personality disorders from the DSM-IV-R [19]. All statements can be scored on 3-point scales ranging from 1 (‘true’), 2 (‘I do not know’), to 0 (‘false’). In some item cases, also the option “not applicable” was offered, e.g., after the item: 'I was absent at work for more than 30 days without a reason”, which cannot be answered if the respondent was unemployed. For the purpose of the current study we counted the number of “true” responses and only used the Anti-Social 14-item subscale measuring a pattern of irresponsible and antisocial behavior since the age of 15. Examples of scale items are “In the past 5 years…” “..I have repeatedly neglected my financial responsibilities”, “..I have often harassed or intimidated other people”, and “..I am very irritable and aggressive and I have repeatedly hit someone or caused someone physical injury in some other way”. The reliability of the Anti-social behavior subscale as assessed with Cronbach’s α was reported to be .78 [89], and was .52 in the present study. The low reliability of the scale will decrease the statistical power of tests of effects on this scale. However, high sample size compensates for low reliability, which is nicely put by Ellis [90] (p.18): ‘Thus, for the purpose of significance testing, any positive reliability is high enough, provided that enough subjects can be added to the research.’ Since our sample size is extremely high, statistical power to detect small or larger effects on this scale is still approaching 1, even though its reliability is just .52.

### Statistical analyses

Preliminary analyses included computing means, standard deviations, and Cronbach’s alphas for all variables under study, and bivariate correlations between relevant variables. We used the sum score for the anti-social behavior subscale, and mean item scores for all other measures. As a first step of our analyses we computed *t*-tests assuming unequal population variances to verify expected relationships between sex, mental disorder indices, and the three autonomy-connectedness components. Cohen’s *d* was calculated assuming equal population variances.

Missing values were dropped from mediation and moderation analyses case-wise guaranteeing that the total effect of sex was equal to its direct effect plus the indirect effects via autonomy components. Because the number of missing values was only very small (28, or 0.5% of the total sample size), we did not apply multiple imputation to deal with the missing values. Mediation analyses were conducted using regression analysis. According to Baron and Kenny [91] mediation occurs when three conditions are met (see Fig 1): 1) a significant association between the predictor and mediator exists (*a*); 2) a significant association between the mediator and the outcome variable (controlled for the predictor) exists (*b*); 3) the relationship between the predictor and the outcome variable (*c*) is substantially reduced when controlling for the mediator (*c’ = c-ab)*. A first series of regression analyses consisting of three steps was carried out to conduct the mediation analyses. First, background variables age (in years), relationship status, and education (treated as a continuous variable) were entered in the analyses. Sex was entered secondly, and SO, SA, and CMNS were entered thirdly. These three steps provided the *c*, *b*, and *c’* values of Fig 1. A second set of regression analyses was carried out to obtain the direct effects of sex on autonomy-connectedness (*a* in Fig 1). In these analyses, one autonomy-connectedness component was the outcome variable, and the background variables and sex were the predictor variables. Sobel *z*-values were carried out to test the mediation by autonomy-connectedness components. We choose to carry out the mediation analyses using the Baron and Kenny methodology and Sobel tests for two reasons. First, of all methodologies, these were best known. Second, although we were aware that other methodologies such as bootstrapping and ‘distribution of the product’ could be considerably more powerful (e.g., [92]), the statistical power detecting mediation in our study with the Sobel test was approaching 1 anyway, even for small effects, because of the very large sample size. Effect sizes as R^2^ change were interpreted using Cohen’s rules of thumb for small (1%), medium (6%), and large (14%) effect sizes.

The moderation effects were tested by adding interaction effects in the fourth step of the first series of regression analyses. To examine if sex moderates the effect of autonomy on disorder characteristics, the three interactions of sex with autonomy components were added simultaneously. Alternatively, to examine autonomy patterns we instead added the three interactions between autonomy components in the fourth step. Prior to the moderation analyses the autonomy-connectedness scales were centered. Simple slope analysis was applied to interpret a significant interaction [93]. For interactions between the continuous autonomy components we examined the simple slopes at +1 *sd* and -1 *sd* from the mean of either SA or CMNS (see [93] pp. 14–22, for an example).

All analyses were conducted using SPSS 19.0. Because we carried out all analyses on each of six outcome variables, we used the more conservative significance level of .01 rather than .05 in all our regression and mediation analyses.

## Results

### Preliminary analyses

Means, standard deviations and Cronbach’s alpha’s for the variables under study for the whole sample (N = 5,525) are shown in Table 1.

**Table 1:** Cronbach’s alpha values, means and standard deviations^1^.

*Scale*	*Mean(SD)*		*Total(SD)*	*N*		*Cronbach’s Alpha*^2^
	*Men*	*Women*		*Men*	*Women*	
**ACS-30**						
Sensitivity to others	3.05 (.45)	3.36 (.45)	3.21 (.48)	2580	2930	.78
Self-awareness	3.91 (.65)	3.72 (.71)	3.81 (.69)	2580	2930	.76
Capacity for Managing New Situations	3.13 (.80)	2.91 (.80)	3.06 (.80)	2580	2930	.74
**Eating disorders**						
Body Dissatisfaction	2.55 (.95)	3.45 (1.23)	3.02 (1.19)	2580	2924	.90
Bulimia	1.42 (.54)	1.64 (.73)	1.54 (.66)	2580	2924	.86
Thinness	2.13 (.85)	2.71 (1.02)	2.44 (0.99)	2580	2924	.84
**Anxiety**	1.31 (.46)	1.42 (.55)	1.37 (.51)	2578	2922	.91
**Depression**	1.22 (.25)	1.27 (.28)	1.25 (.27)	2586	2939	.85
**Anti-social personality**	.55 (1.00)	.29 (.71)	.42 (.87)	2586	2939	.52

^1^ All sex differences are statistically significant (*p* < .001).

^2^ Based on the data set without missing values (*N* = 5497).

Effects of sex on autonomy-connectedness components were according to expectations: Women scored higher on SO (*t* (5438.4) = -27.76, *p <* .001, Cohen’s *d* = -.70), whereas men scored higher on SA (*t* (5502.5) = 10.76, *p <* .001, *d* = .29) and CMNS (*t* (5421.8) = 9.98, *p <* .001, *d* = .27). Also according to expectations was that women reported significantly higher levels of depression (*t* (5523.0) = -7.32, *p <* .001, *d* = -.20), anxiety (*t* (5478.9) = -8.49, *p <* .001, *d* = -.23), drive for thinness (*t* (5481.5) = -23.10, *p <* .001, *d* = -.62), bulimia (*t*(5354.6) = -13.16, *p <* .001, *d* = -.35) and body dissatisfaction (*t* (5415.0) = -30.52, *p <* .001, *d* = -.81). Also as expected, men scored significantly higher on anti-social behavior (*t* (4586.9) = 11.19, *p <* .001, Cohen’s *d* = .31). Noteworthy were the very low sum scores on the antisocial behavior scale, with 78.4% of the women and 65.0% of the men having score zero.

Bivariate correlations between variables are presented in Table 2. Confirming results from previous research (e.g., [28]) SA and CMNS were positively correlated with each other and negatively with SO. SO was positively related to the internalizing disorder indices (.20 to .28) and negatively to anti-social behavior (-.12). SA, on the other hand, was negatively related to all disorder indices (-.05 to -.31). CMNS was also negative related to all internalizing disorder indices (-.17 to -.29), but positively to anti-social behavior (.03). The correlations of the three autonomy-connectedness scales with anti-social behavior were small (up to .12), and medium (.17 to .31) with the internalizing disorder indices, indicating that: (i) autonomy-connectedness explained individual differences in disorder indices, but (ii) played a lesser role in explaining anti-social behavior.

**Table 2:** Bivariate Pearson correlations between outcome and predictor variables.

Scale	1	2	3	4	5	6	7	8	9
1. Sensitivity to others	1	-.33	-.27	.26	.21	.22	.28	.22	-.12
2. Self-awareness		1	.38	-.18	-.27	-.21	-.30	-.31	-.050
3. Capacity for Managing with new situations			1	-.16	-.15	-.20	-.25	-.29	.030^#^
4. Drive for thinness				1	.55	.65	.25	.27	-.010^#^
5. Bulimia					1	.46	.36	.29	.12
6. Body dissatisfaction						1	.19	.24	-.005^#^
7. Anxiety							1	.62	.17
8. Depression								1	.17
9. Anti-social behavior									1

^#^ Not statistically significant at p <.01

Among the disorder indices, the eating disorder indices were strongly correlated to each other (.46 to .65), and depression and anxiety were strongly correlated as well (.62). Correlations between anxiety and depression on the one hand and the eating disorder indices on the other hand were mostly medium (.19 to .36), whereas correlations with antisocial behavior varied from around zero (drive for thinness and body dissatisfaction) to rather small (.17 for depression and anxiety).

### Effects of demographics, sex, and autonomy components on mental health indices

The regression analyses were carried out on 5,497 cases after deleting the 28 cases having incomplete data on sex, disorder indices, or the ACS-30. The results of the regression analyses are presented in 3. Age, relationship status and education explained between 1.1% and 5.8% of the variance in disorder severity, representing a small to medium effect size. Table 3 shows the effects of each of the demographic variables in the final model (including also the effects of sex and autonomy-connectedness). Age was positively related to depression and drive for thinness, and negatively related to anxiety, bulimia, body dissatisfaction and anti-social behavior. Singles reported higher levels of depression, anxiety, and anti-social behavior, compared to participants in a relationship. Finally, higher education was associated with lower scores on all disorder indices, with the exception of bulimia and body dissatisfaction for which no association was found.

**Table 3:** Mediation analysis analyses with sex, mediators SO (sensitivity to others), SA (self-awareness) and CMNS (capacity for managing new situations), and the disorder indices as outcome variables. Unstandardized regression coefficients (B) are reported with their standard errors (SE).

	Depression B(SE)	Anxiety B(SE)	Thinness B(SE)	Bulimia B(SE)	Body dissatisfaction B(SE)	Anti-social behaviour B(SE)
**Step 1**^1^						
Age	.010 (.002)	-.029 (.004)	.020^#^ (.008)	-.082 (.005)	-.004 (.001)	-.079 (.001)
Relationship	-.063 (.008)	-.087 (.016)	-.052^#^ (.031)	-.054 (.021)	-.009^#^ (.036)	-.104 (.022)
Education	-.034 (.007)	-.042 (.014)	-.128 (.028)	-.011^#^ (.019)	-.068^#^ (.033)	-.089 (.020)
R^2^	.022	.023	.008	.058	.011	.041
**Step 2**						
Sex^2^	.048 (.007)	.106 (.014)	.579 (.027)	.214 (.018)	.899 (.032)	-.251 (.019)
R^2^	.031	.034	.093	.084	.150	.074
ΔR^2^	.009	.011	.085	.026	.138	.034
**Step 3**						
SO	.030 (.004)	.086 (.008)	.145 (.015)	.055 (.010)	.050 (.018)	-.061 (.011)
SA	-.057 (.004)	-.060 (.007)	-.078 (.015)	-.114 (.010)	-.093 (.017)	-.057 (.010)
CMNS	-.042 (.004)	-.077 (.009)	-.047 (.015)	-.039 (.010)	-.143 (.017)	.011^#^ (.010)
Sex	-.0002^#^ (.007)	.003^#^ (.014)	.443 (.028)	.133 (.018)	.798 (.033)	-.221 (.020)
R^2^	.170	.156	.133	.142	.181	.084
ΔR^2^	.139	.121	.041	.058	.033	.010

^1^ Effects are for the final model;

^2^Sex (0 = male, 1 = female)

^#^ Not statistically significant at *p* <.01

After controlling for demographics, sex had a small effect on depression and anxiety (improved explained variances of 0.8% and 1.0%, respectively), small to large effects on eating disorder indices (2.4% to 8.3%), and a small to medium effect on anti-social behavior (2.9%). Men had higher scores on anti-social behavior, whereas women had higher scores on all other disorder indices. Controlling for demographic variables and sex, the autonomy components improved the prediction of all disorder indices in step 3 of the sequential regression analyses. The additional explained variance of the autonomy components was 12.7% for depression, 11.9% for anxiety, which are medium to large effect sizes, and 4.3% for drive for thinness, 5.8% for bulimia, 3.6% for body dissatisfaction and 1.2% for anti-social behavior, which are small to medium effect sizes (all *p* < .001, see last row of Table 3). For disorder indices depression, anxiety, thinness, bulimia, and body dissatisfaction we found the same pattern; a positive association with SO, and negative associations with SA and CMNS. For anti-social behavior we found a negative association with SO and SA, and no association with CMNS.

### Mediation analyses

Table 4 summarizes the results of the mediation analyses. The first row presents the total effects of sex on the disorder indices after controlling for the demographics (all *p* < .001), i.e., the effects of sex in step 2 of the previously discussed regression analyses. This total effect of sex is decomposed into the direct effect of sex (second row, also presented in Table 3) and the mediated effects of the autonomy-connectedness components (rows three to five). The mediated effects were obtained by multiplying the effects of sex on autonomy-connectedness after controlling for demographics (not shown in tables) by the effects of autonomy-connectedness on the disorder indices (presented in Table 3). We expressed direct and mediated effects as a percentage of the total effect, an effect size measure of mediation that works well for large sample sizes ([92], p.85).

**Table 4:** Total effects of sex, direct effects of sex, and effects of sex mediated by autonomy components. The mediated effects are tested using the Sobel z test.

	Depression.	Anxiety	Thinness	Bulimia.	Body dis-satisfaction	Anti-social pers. disorder
Total effect	.048	.106	.579	.214	.899	-.251
Direct effect	-.0002^#^	.003^#^	.443	.133	.798	-.221
Mediated by SO	.018 z = 7.55	.060 z = 10.36	.101 z = 9.05	.038 z = 5.38	.035 z = 2.76	-.042 z = 5.42
Mediated by SA	.016 z = 8.36	.017 z = 6.26	.022 z = 4.62	.032 z = 7.55	.026 z = 4.81	.016 z = 4.96
Mediated by CMNS	.012 z = 7.44	.022 z = 7.19	.013 z = 2.99	.011 z = 3.64	.040 z = 6.46	-.003^#^ z = 1.09

^#^ Not statistically significant at *p* <.01

Note SO = sensitivity to others; SA = self-awareness; CMNS = capacity for managing new situations

The effect of sex on depression and anxiety was completely mediated by autonomy-connectedness; the effect of sex was very small and no longer significant after controlling for autonomy-connectedness. The effect of sex was mediated by all three components (all Sobel tests were highly significant), with most of the effect mediated by SO (40% for depression, 59% for anxiety), and less but still substantially by SA (31% and 25%) and CMNS (25% and 17%).

For the eating disorder indices we found the same pattern of results. Most of the effect of sex was not mediated by autonomy-connectedness (63% for bulimia, 76% for thinness, and 88% for body dissatisfaction), but all three components mediated part of the effect. SO explained the largest part of the effect for bulimia and thinness (17%), and CMNS for body dissatisfaction (4.3%). Most of the effect of sex on anti-social behavior was also not mediated (85%). SO mediated 20% of the effect of sex, whereas CMNS did not mediate the effect. Interestingly, mediation by SA was inconsistent, i.e., the effect of sex on anti-social behavior *increased* after controlling for SA.

### Moderation analyses

The upper half of Table 5 presents the results on the three sex × autonomy moderation effects. The combined effect size of the interactions was very small, as evidenced by the small increase in explained variance (never more than 0.3%). Only the prediction of depression, drive for thinness, and bulimia was improved by the three interactions. If there was a significant interaction, the effect of the autonomy component on the disorder index was always slightly stronger for women. More specifically, the effect of SA on bulimia was negative and significant for men, but stronger for women. Finally, no effect of SA on drive for thinness was observed for men (*b* = -.043, *t* = -1.36, *df* = 5486, *p* = .17), and a negative effect of SA for women.

**Table 5:** Sex × Autonomy and autonomy× autonomy interaction effects on depression, anxiety, thinness, bulimia, body dissatisfaction, and anti-social behavior. Unstandardized regression coefficients are reported.

	Depression B(SE)	Anxiety B(SE)	Thinness B(SE)	Bulimia B(SE)	Body dissatisfaction B(SE)	Anti-social behaviour B(SE)
Sex^1^ × Autonomy						
Sex × SO	.017 (.008)	.025 (.015)	.042 (.030)	.016 (.020)	.066 (.035)	-.001 (.021)
Sex × SA	-.019 (.008)	-.018 (.015)	-.097* (.030)	-.057* (.020)	-.021 (.035)	.022 (.021)
Sex × CMNS	.008 (.007)	.008 (.015)	.022 (.029)	.004 (.019)	-.008 (.035)	.005 (.021)
ΔR^2^	.002*	.001	.003*	.002*	.001	.0003
Autonomy x Autonomy						
SO × SA	-.020* (.003)	-.024* (.007)	-.003 (.014)	.001 (.009)	-.019 (.016)	.004 (.010)
SO × CMNS	.004 (.003)	-.011 (.007)	-.019 (.014)	-.006 (.009)	-.008 (.016)	-.004 (.010)
SA × CMNS	.009 (.003)	.007 (.012)	-.029 (.014)	-.014 (.009)	-.027 (.016)	-.011 (.010)
ΔR^2^	.010*	.006*	.001	.0005	.001	.0003

^1^Sex (0 = male, 1 = female);

*p < .01.

Note SO = sensitivity to others; SA = self-awareness; CMNS = capacity for managing new situations

The lower half of Table 5 presents the results on the interactions of the autonomy components. The three interactions only improved the explained variances of depression and anxiety. The combined effect sizes of the interactions were again small, with an increase in explained variance of 1.0% and 0.6% of depression and anxiety, respectively. With respect to depression, the SO × SA interaction below average (*b* = .095, *p* < .001), at average (*b* = .058, *p* < .001), and one *SD* above average (*b* = .021, *p* = .021). With respect to anxiety, the effect SO on anxiety decreased in SA (*b* = -.07, *p* < .001). Simple slope analyses revealed, however, that the effect of SO on anxiety was negative for SA one *SD* below average (*b* = .22, *p* < .001) to SA one *SD* above average (*b* = .12, *p* < .001)). To conclude for all simple slope analyses for depression and anxiety, the interaction effects were small and the direction of the effects was not affected by the interactions.

### Additional exploratory analyses

We ran one additional regression analysis to find a potential explanation for the differences in the mediation results: Why did autonomy fully mediate the sex difference in depression and anxiety, but not the sex difference in the eating disorder indices? One explanation was that other than autonomy-related mechanisms mediated the effect of gender on eating disorders as well. For instance, Stice’s dual pathway model on bulimic behavior [94] assumed that body dissatisfaction is the result of the pressure to be thin, and that bulimic behavior is partly due to the pressure to be thin and to body dissatisfaction. Therefore we tested if drive for thinness partially mediated the sex difference in body dissatisfaction, and if both drive for thinness and body dissatisfaction partially mediated the sex difference in bulimia.

Adding drive for thinness to the final model of the sequential regression analyses (of which the results are presented in Tables 3 and 4) on body dissatisfaction, showed a further mediation of the sex difference in body dissatisfaction; the sex difference of .782 (penultimate column of Table 4) was reduced to .475, which is a further reduction of the total effect of sex by 35%. This sex difference was still significant, however, hence mediation was still partial. Adding both thinness and body dissatisfaction to the model explaining bulimia resulted in a reversal of the sex difference. The sex difference was .130 (fourth column of Table 4), but after controlling for thinness and body dissatisfaction the sex difference was -.058 (*p* < .001). That is, men scored higher on bulimia than women, after controlling for demographics, autonomy-connectedness, thinness, and body dissatisfaction. To summarize the additional exploratory analyses, also after taking predictors of eating disorders into account, the effect of sex on eating disorders was not fully mediated.

## Discussion

The main aim of the present study was to examine to what extent autonomy-connectedness components—self-awareness, sensitivity to others, and capacity for managing new situations—would mediate the relationship between being male or female and several clusters of symptoms of psychopathology, in particular severity of depression, anxiety, bulimia, drive for thinness, body dissatisfaction and the anti-social personality disorder. We tested our hypotheses in a large, representative community sample, which minimized selection bias mechanisms common in clinical samples.

Our results confirmed the sex differences in autonomy-components and symptoms of psychopathology. The autonomy-connectedness components explained part of psychopathology, also after controlling for demographics and sex, with strongest associations with depression and anxiety (medium to large). Directions of effects were largely as expected. For the internalizing disorder indices (depression, anxiety, drive for thinness, bulimia, body dissatisfaction) we found the same pattern, i.e. a positive association with SO, and negative associations with SA and CMNS. For anti-social behavior we found a negative association with SO and SA, and no association with CMNS. Our main finding was that the three autonomy components together fully mediated the effect of sex on depression and anxiety symptom severity, and partly on symptom severity of eating disorder (drive for thinness, bulimia, and body dissatisfaction) and the anti-social personality disorder, with SO generally being the strongest mediator. Our moderation analyses revealed very small to small interaction effects, with stronger effects of some autonomy components on symptoms of psychopathology for women, and interaction effects of autonomy components on anxiety and depression, although these interaction effects did not affect the direction of the main effects.

These results confirmed the theories and findings discussed in the introduction of this paper, and strongly suggested that autonomy plays an important role in explaining symptoms of psychopathology and sex differences therein. Particularly, our findings that autonomy components explained a substantial part of depression and anxiety symptomatology, and completely mediated the sex differences in depression and anxiety, suggested that autonomy is essential in understanding, and may even be vital for the treatment of these two disorders. Autonomy-connectedness components likely also play a role in explaining symptomatology of eating disorders and the antisocial personality disorder and sex differences therein. Mediation was not complete, indicating that additional mechanisms than (sex differences in) autonomy-connectedness solely account for sex differences in the eating disorders and the antisocial personality disorder. Incorporating body dissatisfaction and drive for thinness in the mediation model of bulimia, which was in line with Stice’s model, did also not result in complete mediation. Perhaps future research could take other factors such as alexithymia and impulsivity into account as well [61].

Finally, interaction effects being either absent or only very small to small suggested that autonomy patterns or combinations had minor importance in explaining symptomatology; effects of autonomy components were mainly additive and linear.

Theoretically, the current study, together with previous studies on autonomy-connectedness, provided robust evidence for Bowlby’s [21, 22] idea that poor autonomy, in our terms: poor autonomy-connectedness [28] is a serious risk factor for psychopathology. More at risk to suffer from mental disorders are plausibly those who lack the valid, reliable, adult “inner compass” for self-steering we labelled autonomy-connectedness, i.e., those who are insufficiently aware of their needs, have no or only limited access to their own wishes and opinions, are under- or overly involved with needs, wishes and opinions of others, and/or hardly able to manage new situations. Simultaneously, our findings agreed with neo-analytical object-relational perspectives addressing gender-identity development. Poor autonomy-connectedness namely showed a sex-specific nature, with low sensitivity to others predicting psychopathology more prevalent in men (externalizing disorders, antisocial behaviour), and high sensitivity to others predicting mental disorders with higher prevalence in women (internalizing disorders). This supported our idea [27, 31, 40] that mental disorders occurring more in men reflect “extreme separation and denial of attachment”, and those more typical for women “extreme connectedness”, extreme manifestations of preoccupation with others. Regarding self-awareness and capacity for managing new situations one could argue “the more the better” for mental health. Theoretically, the finding that autonomy-connectedness components were strongly connected to all mental disorder indices under study, suggested that very different disorders like bulimia and anti-social personality disorder may have some common ground, namely in deficient autonomous functioning, albeit in different ways.

Did our observed associations between autonomy-connectedness and mental disorders imply causation of mental disorders by autonomy-connectedness? Our cross-sectional study did not allow us to draw conclusions on causal or temporal relationships between the variables under study. Note, however, that sex necessarily preceded all other variables, and that, theoretically, autonomy-connectedness most plausibly preceded mental health disorder symptoms, which indicated the validity of the causal model (Fig 1). We should also keep in mind that experimental manipulation of most variables under study is impossible, among others because of ethical concerns. It is surely (also) possible that direction of causation between autonomy-connectedness and types of psychopathology flows from the latter to the former. For example, deficits in ability to socialize due to autism or preoccupation with own psychopathology symptoms might contribute to failure to develop autonomy-connectedness. In addition, (also links to) third variables may play important roles. Hence, we cannot conclude from the results from our cross-sectional study to what extent autonomy-connectedness causes psychopathology, to what extent the other way around is true, or to what extent other, related variables contribute to the associations found; even though our model of Fig 1 seems to be line with our results (see also Strengths and limitations below).

Until now autonomy problems were mainly associated with internalizing disorders. Our study expanded several previous studies (e.g., [48, 95]) in showing that autonomy problems might be more widespread, also being related to characteristics of the anti-social personality disorder, thus, of externalizing disorders. Indices of the anti-social personality disorder were also characterized by low self-awareness; this is relevant because individuals with this disorder are known to be especially expressive regarding what they want and desire, particularly the more (instrumentally) aggressive ones (e.g., [96]). Apparently being expressive and externalizing does not automatically imply high awareness of own internal states. According to the traditional definition of autonomy these individuals would be described as being high in autonomy, i.e., they do what they like to do irrespective of others or social norms. However, studies among adolescents showed that engaging in antisocial behavior is related to adolescents blocked access to adult options of autonomous decision making ([97]; see also [98]), hampering the development of a healthy self. We believe that high autonomy-connectedness is not possible without high self-awareness, so we are inclined to conclude that the anti-social personality disorder is also rooted in low autonomous functioning. These individuals probably experience or wish low connectedness to others while at the same time having very little insight into their own internal states rendering them vulnerable and socially isolated. In the same vein individuals with internalizing disorders characteristics like depression or bulimia experience or wish a lot of connectedness with others, perhaps too much, making them overly dependent on others. They also lack self-insight, rendering them equally vulnerable and socially isolated. One could argue that avoiding and overly seeking social connectedness without self-awareness are two problematic sides of the same coin.

Theoretically, our study demonstrated that examining sex differences (and this is likely also the case for differences along other diversity dimensions, such as in age or ethnicity) can be a useful strategy for obtaining insights into psychological phenomena, in this case psychopathology and mental health, that cannot be easily acquired in other ways (see also, [8, 12]). Rutter, Caspi and Moffit [8] formulated three criteria for potential proximal risk or protective factors for being implicated in causal mechanisms predisposing to psychopathology. To explain sex differences, these proximal factors must meet: (1) evidence that the risk factors do indeed differ between males and females; (2) evidence that within each sex they provide risk for or protection against particular mental disorders; and (3) evidence that when their effects are included in a causal model, they either reduce or eliminate the sex difference in the psychopathology being studied. Moreover, they added that it would, ultimately, be necessary to determine how the links across the three levels are mediated. Rutter, Caspi and Moffit [8] concluded that no variables had yet met all three criteria. Our study showed that autonomy-connectedness did satisfy the three criteria and may therefore be a good candidate.

### Strengths and limitations

We tested our hypotheses in a large, representative community sample, enabling observation of the full range of possible scores of both autonomy and symptomatology, which minimizes selection bias mechanisms common in clinical samples. Despite this strength, there were, aside from the study’s inability to provide conclusive insights into the temporal and/or causal links between the variables (see above), some other limitations as well. First, there is a difference between disorder symptomatology and diagnoses of the disorders. Hence, although our results concerning sex differences in disorder indices were completely in line with the well-known sex differences in prevalence of these disorders, our mediation results on disorder symptomatology did not necessarily imply mediation of sex differences in disorder prevalence.

Second, we solely used self-report measures for autonomy-connectedness and psychopathology indices. This means that social desirability or other biases and sex differences therein may have confounded our results. In fact, findings might (partly) be due to participants answering according to gender role stereotypes or negativity tendencies. Another limitation could be that we used regression analysis rather than structural equation modelling, and the Baron and Kenny methodology and the Sobel test rather than testing mediation using bootstrapping. Note, however, that we used traditional regression analysis and mediation methodology because they are better known, and because both the traditional and modern methodology have very high power in very large sample sizes like ours. A final limitation concerned the use of one of our measures, the VKP. The majority of respondents scored zero on the VKP; this pointed to the possibility that the scale measured more extreme symptoms of anti-social behavior, indicating its higher usefulness for a clinical sample than a representative, population sample.

### Suggestions for future research

Our first suggestion is testing the mediation model using observational measures of autonomy-connectedness (if possible) and/or symptom reports by significant others or clinicians, e.g., SCID scores. Regarding the last point it is important to note that, in this study, sex had an, although statistically significant, somewhat small effect on depression and anxiety symptom reporting. Several authors (e.g., [99]), for example, warned that underreporting of depressive symptoms might generally be a problem in epidemiological studies as well as clinical practice, and that this might specially be true for men. It would be interesting and relevant to further examine the role of autonomy-connectedness using other or additional indices of mental disorders symptoms or measurement conditions. Regarding the issue of causality, an indication can be obtained by experimentally strengthening (poor) autonomy-connectedness and examining to what extent individuals then recover from mental disorders. However, also in that case, the issue if autonomy had been the causal factor would remain inconclusive. As autonomy-targeted interventions, particularly for groups, have been well developed and documented [100–102], randomized clinical trials for evaluating their effects for well-defined clinical populations seem the preferred way. In addition, we recommend possible (quasi-)experimental study set-ups, or longitudinal studies as future directions.

In addition, we recommend including other autonomy-related concepts to examine how these concepts, together with autonomy-connectedness, account for disorder symptomatology and sex differences therein. An example is rumination, and other ones are alexithymia and impulsivity [61]. Incorporating these other concepts are necessary in order to fully explain sex differences in eating disorders and the antisocial personality disorder.

An additional interesting line of future research is to look closer into the sensitivity to others part of autonomy. This component is currently defined in a rather general way as (self-reported) sensitivity to others’ needs, opinions and wishes. It would be interesting to see whether men and women respond differently in terms of their sensitivity to *different kinds* of opinions. For example, women’s self-reports might reveal more sensitivity to subjects dealing with outer appearance, whereas men’s might reflect more sensitivity to subjects dealing with toughness and social status. In addition, such possible differences might also be examined using observational measures. This could add to explaining why more women develop eating disorders and more men anti-social personality disorder.

### Clinical implications

As autonomy-connectedness reflects a rather basic psychological capacity, self-governance while (well-) connected to others, we believe therapy should focus more on restoring and/or developing this capacity. The perspective that therapy should be more targeted at underlying deficits of mental disorders than on symptoms fits well with the more recent definitions of mental disorders of DSM-5 [18], which emphasized underlying deficits in psychological functioning as a core characteristic. Targeting therapy more at autonomy-connectedness may also be useful for the more attachment-oriented therapy types such as Schema-Focused Therapy and Emotion-Focused Therapy. Recent studies showed only moderate associations of autonomy-connectedness with other attachment-related concepts that are core concepts within these therapy types [103], such as insecure attachment styles (e.g., anxious and avoidant attachment) and Early Maladaptive Schema’s (EMS; pervasive, negative perceptions of oneself, others, and the environment, that give meaning to each experience [104, 105]). The moderate associations indicated that the three sets of concepts were related, as one would expect, but that the capacity for self-governance while connected to others was a separate factor. For therapy purposes, EMS help identifying the predominant *domains* of psychological suffering; the insecure attachment styles reflect the dominant *coping* with the experienced attachment insecurity; and autonomy problems reflect the deficits in the self(-image) and the psychological *self-steering capacity* while (trying to be) connected to others. To the latter belong the emotions that are not (sufficiently) felt, the needs that are unrecognized, the individual wishes, preferences, opinions that still have to be detected and developed. All these concepts seem relevant for attachment and recovery from insecure attachment experiences, and therapy might profit from using them side by side and flexibly together instead of narrowing the focus to only one of them.

Since the sex differences in depression and anxiety were fully mediated by autonomy, our results indicated that men who have low autonomy are equally at risk to develop depression and anxiety as women with similar autonomy. Clinicians could benefit from these findings by focusing more on autonomy problems in patients with depression and anxiety. Especially promoting a healthy balance between sensitivity to others and self-awareness may be beneficial for these patients. For patients with eating disorders and the anti-social personality disorder, strengthening autonomy may be beneficial as well, although women are still more at risk for eating disorders and men for the anti-social personality disorder given similar autonomy-connectedness scores. Finally, it is important to note that strengthening autonomy implies something different when it comes to patients with the anti-social personality disorder. These patients probably have low sensitivity to others (together with low self-awareness), thus autonomy promoting therapy should focus on *enhancing* sensitivity to others and self-awareness, while for the internalizing disorders the focus should be on tempering sensitivity to others (and enhancing self-awareness as well). All in all it seems that sensitivity to others together with self-awareness and capacity for managing new situations are key to promoting healthy autonomous functioning regarding all disorders currently studied.

Enhancing autonomy-connectedness can be done within Autonomy Enhancing Treatment (AET), a transdiagnostic form of therapy that is mostly offered as a group treatment [100], but can also be provided as individual treatment; it moreover can be added to other psychotherapy types but is also offered as stand-alone treatment. The main aim of AET is enhancing AC, more in particular, strengthening Self-awareness (SA) and Capacity for managing new situations (CMNS) and regulating Sensitivity to others (SO). Participants work on their own personal, autonomy related goal(s). For many women, AET therapy goals often concern getting more into contact with, and learning to make decisions more congruent with their own wishes and needs, whereas for men becoming aware of their vulnerability and dependency needs, and expressing these in social interactions, thereby overcoming masculinity norms, may be more important AET therapy goals. AET has recently been protocolled [100]. In mental health care, AET—guided by two therapists—comprises 15 sessions that are divided into three main stages. During the introductory stage, personal autonomy-related goals are specified. In the second phase, patients learn to develop (higher) SA and CMNS and a more regulated SO, by reflecting, group discussions and group exercises. Among themes discussed during the meetings are “survival strategies”; learning history; parent-child relationship and role models; the development, nature and adaptiveness of schemas; setting boundaries; communication; relationships, sexuality and intimacy; functioning at work. As male and female patients together participate in one group, patients are at some point offered the opportunity to discuss certain topics (e.g., sexuality) in subgroups (men and women separately), which is enabled by the availability of the two therapists. The ending stage consists of consolidation of accomplished goals and relapse prevention. Every session one patient is appointed as the chair(wo)man, which further boosts patients’ autonomy-connectedness. Next to being gender-sensitive, AET is diversity sensitive more in general. Targeting the person behind the symptoms, AET naturally touches (ethnic/cultural) identity.

In summary, clinical implications of the current research were that treatment of depression, anxiety, but also eating disorders and antisocial personality disorder might benefit from (adding) a stronger focus on autonomy strengthening in patients. In addition, therapists should be aware that the direction of such focus should depend upon their patient’s specific autonomy; the challenge is to find the healthy balance between sensitivity to others and self-awareness by lowering or increasing the first and developing and strengthening the latter.

## Supporting information

S1 Appendix Questionnaire instructions and items. (DOCX)
