# Supplementary material for: Autonomy-connectedness mediates sex differences in symptoms of psychopathology
Source: PLoS One. 2017 Aug 3;12(8):e0181626. doi: 10.1371/journal.pone.0181626 (PMC5542470; doi:10.1371/journal.pone.0181626)
Supplement: S1 Appendix — (DOCX) [file pone.0181626.s001.docx]

**Appendix Questionnaire Instructions and Items**

Part 1

This first part of the questionnaire entails a number of statements that have been grouped together. Please, read every group of statements carefully. Then, chose, in each group, the statement that best reflects how you felt in the past week, including today.

Take care that you first read all statements of a group before making your choice.

1.

0 I do not feel sad.

1 I feel sad

2 I am sad all the time and I can't snap out of it.

3 I am so sad and unhappy that I can't stand it.

2.

0 I am not particularly discouraged about the future.

1 I feel discouraged about the future.

2 I feel I have nothing to look forward to.

3 I feel the future is hopeless and that things cannot improve.

3.

0 I do not feel like a failure.

1 I feel I have failed more than the average person.

2 As I look back on my life, all I can see is a lot of failures.

3 I feel I am a complete failure as a person.

4.

0 I get as much satisfaction out of things as I used to.

1 I don't enjoy things the way I used to.

2 I don't get real satisfaction out of anything anymore.

3 I am dissatisfied or bored with everything.

5.

0 I don't feel particularly guilty

1 I feel guilty a good part of the time.

2 I feel quite guilty most of the time.

3 I feel guilty all of the time.

6.

0 I don't feel I am being punished.

1 I feel I may be punished.

2 I expect to be punished.

3 I feel I am being punished.

7.

0 I don't feel disappointed in myself.

1 I am disappointed in myself.

2 I am disgusted with myself.

3 I hate myself.

8.

0 I don't feel I am any worse than anybody else.

1 I am critical of myself for my weaknesses or mistakes.

2 I blame myself all the time for my faults.

3 I blame myself for everything bad that happens.

9.

0 I don't have any thoughts of killing myself.

1 I have thoughts of killing myself, but I would not carry them out.

2 I would like to kill myself.

3 I would kill myself if I had the chance.

10.

0 I don't cry any more than usual.

1 I cry more now than I used to.

2 I cry all the time now.

3 I used to be able to cry, but now I can't cry even though I want to.

11.

0 I am no more irritated by things than I ever was.

1 I am slightly more irritated now than usual.

2 I am quite annoyed or irritated a good deal of the time.

3 I feel irritated all the time.

12.

0 I have not lost interest in other people.

1 I am less interested in other people than I used to be.

2 I have lost most of my interest in other people.

3 I have lost all of my interest in other people.

13.

0 I make decisions about as well as I ever could.

1 I put off making decisions more than I used to.

2 I have greater difficulty in making decisions more than I used to.

3 I can't make decisions at all anymore.

14.

0 I don't feel thatI look any worse than I used to.

1 I am worried that I am looking old or unattractive.

2 I feel there are permanent changes in my appearance that make me look unattractive

3 I believe that I look ugly.

15.

0 I can work about as well as before.

1 It takes an extra effort to get started at doing something.

2 I have to push myself very hard to do anything.

3 I can't do any work at all.

16.

0 I can sleep as well as usual.

1 I don't sleep as well as I used to.

2 I wake up 1-2 hours earlier than usual and find it hard to get back to sleep.

3 I wake up several hours earlier than I used to and cannot get back to sleep.

17.

0 I don't get more tired than usual.

1 I get tired more easily than I used to.

2 I get tired from doing almost anything.

3 I am too tired to do anything.

18.

0 My appetite is no worse than usual.

1 My appetite is not as good as it used to be.

2 My appetite is much worse now.

3 I have no appetite at all anymore.

19.

0 I haven't lost much weight, if any, lately.

1 I have lost more than five pounds.

2 I have lost more than ten pounds.

3 I have lost more than fifteen pounds.

20.

0 I am no more worried about my health than usual.

1 I am worried about physical problems like aches, pains, upset stomach, or constipation.

2 I am very worried about physical problems and it's hard to think of much else.

3 I am so worried about my physical problems that I cannot think of anything else.

21.

0 I have not noticed any recent change in my interest in sex.

1 I am less interested in sex than I used to be.

2 I have almost no interest in sex.

3 I have lost interest in sex completely.

Part 2

Below, you will find 30 statements referring to yourself.

Indicate, for each statement, to what extent you agree or disagree. For each item, there is only one answer. Please, chose the option that first comes to mind.

1 = I totally disagree; 2 = I slightly disagree; 3 = I partly disagree and partly agree; 4 = I slightly agree; 5 = I totally agree

1. I often find it difficult to determine what I really want

2. I am usually able to dismiss the thoughts of someone else’s misery

3. When I have to do something against others’ will I am very restless

4. I hate detachment

5. It is difficult for me to start new activities on my own

6. I often do not know what my opinion about a given subject is

7. I rarely care about others’ feelings and experiences.

8. I find it easy to ignore other people’s comments

9. It is usually obvious to me what I like best

10. If I disagree with others, I express it directly

11. I am rarely occupied with other people’s opinions about me

12. I easily come to grips with a new problem on my own13. If I think of having to say goodbye to a beloved person, I break down in advance

14. If it was up to me , I would spend most of my time in familiar surroundings

15. When I am asked what I want I usually know the answer immediately16. I rarely tend to ask other people for advice

17. I often analyze others’ feelings thoroughly

18. I need a lot of time to get used to new surroundings

19. I often wonder about other people think about me

20. When I take important decisions concerning my life, I do not consider other people’s wishes and opinions

21.I can hardly bear the situations when someone is angry with me

22. Under the influence of others’ opinions I often change my opinion

23. I am a very adventurous person

24. Others’ experiences significantly influence my moods

25. I need others’ advice and guidance very much

26. When I do something that bothers other people I can easily stop thinking about it

27. I quickly begin to feel at ease in new situations

28. I often need love and warmth

29. I can easily back out of things that people who are important to me want me to do

30. I can directly express my opinions on most subjects

Part 3

The questions of this part refer to various opinions, feelings and behaviors. Some of the questions concern food. Other questions concern your feelings about yourself.

Here again there are no good or false answers, sop lease try to answer as honestly as possible.

Read every question and chose the option that best suits you.

1 = never; 2 = seldom; 3 = sometimes; 4 = often; 5 = mostly; 6 = always

I eat when I am upset.

I stuff myself with food.

I have gone on eating binges where I have felt that I could not stop.

1 think about bingeing (overeating).

1 eat moderately in front of others and stuff myself when they're gone.

1 have the thought of trying to vomit in order to lose weight.

1 eat or drink in secrecy.

I think that my stomach is too big.

I think that my thighs are too large.

I think that my stomach is just the right size.

I feel satisfied with the shape of my body.

I like the shape of my buttocks.

I think my hips are too big.

I think that my thighs are just the right size.

I think by buttocks are too large.

I think that my hips are just the right size.

I eat when I am upset.

I stuff myself with food.

I have gone on eating binges where I have felt that I could not stop.

1 think about bingeing (overeating).

1 eat moderately in front of others and stuff myself when they're gone.

1 have the thought of trying to vomit in order to lose weight.

1 eat or drink in secrecy.

Part 4

Below is a list of problems and complaints that people sometimes have. Please read each one care-fully. After you have done so, select one of the numbered descriptors that best describes how much that problem has bothered or distressed you during the past week, including today. Circle the number in the space to the right of the problem and do not skip any items. Use the following key to guide how you respond:

Circle 0 if your answer is NOT AT ALL; Circle 1 if A LITTLE BIT; Circle 2 if MODERATELY; Circle 3 if QUITE A BIT; Circle 4 if EXTREMELY

How much were you bothered the last week by:

1. Nervousness or shakiness inside

2. Trembling

3. Suddenly scared for no reason

4. Feeling fearful

5. Heart pounding or racing

6. Feeling tense or keyed up

7. Spells of terror or panic

8. Feeling so restless you couldn’t sit still

9. Feeling that familiar things are strange or unreal

10. Feeling pushed to get things done

Part 5

Now you arrived at the last part. With your answers to the questions of this part you describe the type of person you are. The questions concern your characteristics during the past 5 years, unless they indicate something different.

Below you will see a number of statements. These statements can be more or less applicable to you. While answering the questions you can chose the following options:

1. true The statement in general fits you well.

In some cases the sentence consists several statements connected by ‘and**’**, e.g.,. ‘I love dogs and cats’. If you love dogs and cats, you answer “true”. If you love dogs but not cats, you answer “not true”.

Some statements entail parts separated by ‘or’. If only a part of the statement is characteristic for you, please do answer “true”. Example: ‘I love dogs and cats’. If you love dogs but not cats, you answer “true”; and if you love cats but not dogs, you also answer “true”. If you neither love cats, not dogs, you answer “not true”.

2. Untrue The statement does generally not apply to you.

3. NA Not applicable. This option can be chosen in only some cases, e.g.: “I have been absent at work without a reason for more than 30 days a year.” You cannot answer if you were not employed. In such cases you chose NA.

I have been arrested several times or have done several things for which I could have been arrested if I would have been caught

1.true 2. untrue

I often ripped someone, used a fake name, or lied if I thought that was good for me or if I had fun in there.

1.true 2. untrue

I was never without a permanent address for more than a month, or travelling around more than one month without it being justified.

1.true 2. untrue

I am very irritable and aggressive and I have beaten or in other ways physically mistreated someone several times

1.true 2. untrue

I drove more than five times under the influence

1.true 2. untrue 3.NA

I was held more than five times by the police for too fast or reckless driving

1.true 2. untrue 3.NA

I was more than five times reckless as regards my own or other people’s safety.

1.true 2. untrue

In the past five years I was absent from my work or education without a good reason for more than 30 days a year.

1.true 2. untrue 3.NA

I quit a job or education at least three times without having a sound plan for what I wanted to do afterwards.

1.true 2. untrue 3.NA

In the past five years I did not work for more than 6 months (and did not go to school either), whereas there was work available.

1.true 2. untrue 3.NA

I have failed to meet my financial obligations several times.

1.true 2. untrue 3.NA

I have failed to financially support my family several times although this was expected from me.

1.true 2. untrue 3.NA

Mostly I don’t feel guilty when I hurt, mistreat, or rob someone.

1.true 2. untrue 3.NA

When I have hurt or wronged someone, I almost always feel guilty.

1.true 2. untrue
